# Supplementary material for: Factors associated with differential seropositivity to Leptospira interrogans and Leptospira kirschneri in a high transmission urban setting for leptospirosis in Brazil
Source: PLoS Negl Trop Dis. 2024 May 17;18(5):e0011292. doi: 10.1371/journal.pntd.0011292 (PMC11139309; doi:10.1371/journal.pntd.0011292)
Supplement: S3 Table — (DOCX) [file pntd.0011292.s003.docx]

Table S3. Bivariate models for seroprevalence of *Leptospira kirschneri* Cynopteri (3522C) and *Leptospira interrogans* Icterohaemorrhagiae (Fiocruz L1-130).

|  | **Cynopteri**  **(3522C)** | | | **Icterohaemorrhagiae**  **(Fiocruz L1-130)** | | |
| --- | --- | --- | --- | --- | --- | --- |
| **Characteristic** | **N** | **OR^1^** | **95% CI^1^** | **N** | **OR^1^** | **95% CI^1^** |
| **Individual** |  |  |  |  |  |  |
| Median Age (years) | 2,600 | 1.03 | 1.02, 1.05 | 2,763 | 1.03 | 1.02, 1.04 |
| Sex | 2,600 |  |  | 2,763 |  |  |
| Male |  | — | — |  | — | — |
| Female |  | 1.07 | 0.59, 1.99 |  | 0.69 | 0.52, 0.91 |
| Ethnicity | 2,562 |  |  | 2,724 |  |  |
| Brown |  | — | — |  | — | — |
| Black |  | 0.86 | 0.47, 1.58 |  | 0.97 | 0.72, 1.30 |
| White |  | 0.36 | 0.02, 1.75 |  | 1.21 | 0.64, 2.11 |
| Others |  | 0 |  |  | 0 |  |
| Education | 2,600 |  |  | 2,763 |  |  |
| 5 or less |  | — | — |  | — | — |
| 5 to 9 |  | 0.6 | 0.26, 1.34 |  | 1.09 | 0.76, 1.57 |
| 10 to 12 |  | 0.75 | 0.35, 1.61 |  | 0.95 | 0.66, 1.38 |
| Higher Education |  | 0.86 | 0.05, 4.40 |  | 0.43 | 0.07, 1.43 |
| Never studied |  | 3.64 | 1.35, 8.98 |  | 1.43 | 0.69, 2.73 |
| Employment | 2,600 |  |  | 2,763 |  |  |
| No |  | — | — |  | — | — |
| Yes |  | 0.87 | 0.45, 1.62 |  | 1.73 | 1.30, 2.29 |
| Risk occupation | 2,600 |  |  | 2,763 |  |  |
| No |  | — | — |  | — | — |
| Yes |  | 0.96 | 0.23, 2.67 |  | 2.72 | 1.81, 3.99 |
| **Risk exposures** |  |  |  |  |  |  |
| Walk barefoot | 2,599 |  |  | 2,762 |  |  |
| No |  | — | — |  | — | — |
| Yes |  | 0.75 | 0.39, 1.39 |  | 0.88 | 0.65, 1.18 |
| Use of boots | 2,597 |  |  | 2,760 |  |  |
| No |  | — | — |  | — | — |
| Yes |  | 0.75 | 0.30, 1.58 |  | 1.22 | 0.86, 1.69 |
| Cleaned sewage | 2,286 |  |  | 2,448 |  |  |
| No |  | — | — |  | — | — |
| Yes |  | 1.3 | 0.44, 3.05 |  | 2.55 | 1.75, 3.65 |
| Sewage contact | 2,599 |  |  | 2,762 |  |  |
| No |  | — | — |  | — | — |
| Yes |  | 1.38 | 0.68, 2.62 |  | 1.4 | 1.01, 1.92 |
| Open sewer | 2,598 |  |  | 2,761 |  |  |
| No |  | — | — |  | — | — |
| Yes |  | 1.32 | 0.71, 2.40 |  | 1.26 | 0.94, 1.69 |
| Access to the house is paved? | 2,600 |  |  | 2,763 |  |  |
| No |  | — | — |  | — | — |
| Yes |  | 1.03 | 0.54, 2.14 |  | 1.03 | 0.74, 1.44 |
| **Wall plastered?** | 2,600 |  |  | 2,763 |  |  |
| No |  | — | — |  | — | — |
| Yes |  | 1.1 | 0.38, 2.57 |  | 1.6 | 1.06, 2.36 |
| **Presence of animals in the household** |  |  |  |  |  |  |
| Presence of Cats | 2,600 |  |  | 2,763 |  |  |
| No |  | — | — |  | — | — |
| Yes |  | 1.89 | 1.01, 3.44 |  | 1.09 | 0.78, 1.50 |
| Presence of Dogs | 2,600 |  |  | 2,763 |  |  |
| No |  | — | — |  | — | — |
| Yes |  | 0.86 | 0.45, 1.57 |  | 0.94 | 0.70, 1.25 |
| Presence of Chickens | 2,600 |  |  | 2,763 |  |  |
| No |  | — | — |  | — | — |
| Yes |  | 1.46 | 0.35, 4.09 |  | 1.71 | 0.96, 2.85 |
| ^1^OR = Odds Ratio, CI = Confidence Interval | | | |  | | |
