# Supplementary material for: Factors associated with differential seropositivity to Leptospira interrogans and Leptospira kirschneri in a high transmission urban setting for leptospirosis in Brazil
Source: PLoS Negl Trop Dis. 2024 May 17;18(5):e0011292. doi: 10.1371/journal.pntd.0011292 (PMC11139309; doi:10.1371/journal.pntd.0011292)
Supplement: Attachment — Submitted filename: Response to Reviewer_Final_DSO_v2.pdf [file pntd.0011292.s006.pdf]

Universidade Federal da Bahia
Instituto de Saúde Coletiva
Rua Basílio da Gama S/N, Canela - 40.110-040 Salvador – Bahia – Brasil

E-mail: fcosta2001@gmail.com
federico.costa@ufba.br

Salvador, February 26th, 2024.

Joseph Vinetz

Editor, PLOS Neglected Tropical Diseases

In re: PNTD-D-23-00442R2

Dear

We thank the reviewers for their thoughtful and constructive comments and suggestions. We are submitting our responses along with a revised manuscript, "Factors associated with differential seropositivity to *Leptospira interrogans* and *Leptospira kirschneri* in a high transmission urban setting for leptospirosis in Brazil", for consideration as a research article to be published in PLOS Neglected Tropical Diseases.

Again, we appreciate your consideration in reviewing our manuscript. Please contact me if there are any questions on the preparation of the revision.

Sincerely,

Federico Costa

Corresponding Author

Instituto de Saúde Coletiva, Salvador, Brazil

Manuscript reference number: PNTD-D-23-00442R2

Date: February 26th, 2024.

Response to Reviewer –

Factors associated with differential seropositivity to *Leptospira interrogans* and *Leptospira kirschneri* in a high transmission urban setting for leptospirosis in Brazil

****

Reviewer 3:

Please edit the submission form to eliminate non-English.

The manuscript continues to need English language corrections. For example, "unplaster" should be "unflustered." For example, "Our data suggests" should be changed to "our data suggest."Please go through the entire manuscript again to correct spelling, word choice/diction and grammar errors. This journal does not have copyeditors to do this task.

Response: Thank you for this comment. We appreciate the reviewer's suggestion and have performed a detailed review of the English throughout the text. The changes are highlighted in yellow.

More importantly, this manuscript continues to appear to conflate serogroup seropositivity with actual infecting serovar. For example, in the abstract, "Our data suggests distinct epidemiological 73 patterns associated with serogroups

74 Icterohaemorrhagiae and Cynopteri within the high-risk urban environment for 75 leptospirosis and with differences of spatial niches." "Future studies must identify the different pathogenic serogroups circulating in low-income areas, and further evaluate the potential role of cats in the transmission of the serogroup Cynopteri in urban settings." What the field really needs is precise identification of the actual infecting Leptospira, either by obtaining an isolate for characterization or sequencing-based identification. Cats involved in leptospirosis transmission? Is this statement an error? It is presented as a conclusion. Perhaps "cats" are a confounder for another variable such as another transmitting species such as rodents. Cats are not thought to be important in leptospirosis ecology; no specific data are presented to support this assertion. As the authors state, "Little is known about the animal and environmental reservoirs for

serogroup Cynopteri." This is true, but statistical associations should be treated in a much more circumspect way.

Response: Thank you for this comment and suggestion. Following the reviewer's suggestion we re-formulated the sentence (lines 89-93 and 411-416)

Generally speaking the manuscript can draw statistical conclusions based on seropositivity diagnosing leptospiral exposure but must refrain from drawing causal inference about the infecting strain, unless data are presented to confirm infecting strain.

The manuscript neither cites not discusses the following seminal manuscripts:

Levett PN. Leptospirosis. Clin Microbiol Rev. 2001 Apr;14(2):296-326. doi: 10.1128/CMR.14.2.296-326.2001. PMID: 11292640; PMCID: PMC88975.

Levett PN. Usefulness of serologic analysis as a predictor of the infecting serovar in patients with severe leptospirosis. Clin Infect Dis. 2003 Feb 15;36(4):447-52. doi: 10.1086/346208. Epub 2003 Jan 29. PMID: 12567302.

Abstract

The diagnosis of leptospirosis is often made using the microscopic agglutination test (MAT), in which live antigens representing >20 serogroups undergo reaction with patient serum samples to detect agglutinating antibodies. Data derived from this assay are often used to infer the identity of the infecting leptospiral serovar or serogroup; however, paradoxical reactions and cross-reactions between serogroups are common. To evaluate the usefulness of this approach, data on culture-proven cases of leptospirosis that occurred in Barbados from January 1980 through December 1998 were reviewed. A total of 151 isolates of 4 serovars were identified. The sensitivity of MAT for the prediction of the infecting serovar was determined. Overall, the predominant serogroup at a titer of >or=100 correctly predicted 46.4% of all serovars isolated. If a titer of >or=800 was used as the cutoff, sensitivity decreased slightly to 44.4%. The overall specificity for all serogroups was 64.8%. Serologic analysis appeared to be of little value for the identification of the infecting serovar in individual cases of leptospirosis in humans. Presumptive serogroup reactivity data should be used only to gain a broad idea of the serogroups present at the population level.

Response: We appreciate the reviewers' comments and suggestions. We have included the indicated citations.

Please add a paragraph dedicated to delineating and discussing the limitations of the present work. It should start out something along the lines of "There are limitations to this work that should be considered in the context of the study design and data obtained....."

Response: Thank you for this comment and suggestion. Following the reviewer's suggestion we have added a paragraph dedicated to delineating and discussing the limitations of the work (lines 393-408).

Information provided regarding serogroup Cynopteri is insufficient for the reader, given the important focus on this serogroup. Most publications citing Cynopteri cite L. interrogans server Cynopteri. What is the basis of L. kirschneri Cynopteri. A comprehensive but concise review of the original isolate of Cynopteri is important as well as information about the use of this serovar/serogroup in the literature.

Response: Thank you for this comment and suggestion. According to renowned references such as Faine 1999 and Levett 2001, the Cynopteri serogroup is part of the species *Leptospira Kirschneri*. The classification of the *Leptospira* genus is complex and can sometimes be confusing. It is possible that in some publications the serovar Cynopteri has been erroneously classified as belonging to *L. interrogans* species. We added to the manuscript about this isolate and references, confirming that it belongs to the *L. kirschneri* species.
