# Supplementary material for: Factors associated with differential seropositivity to Leptospira interrogans and Leptospira kirschneri in a high transmission urban setting for leptospirosis in Brazil
Source: PLoS Negl Trop Dis. 2024 May 17;18(5):e0011292. doi: 10.1371/journal.pntd.0011292 (PMC11139309; doi:10.1371/journal.pntd.0011292)
Supplement: Attachment — Submitted filename: Response to Reviewer_Final_DSO_v6.pdf [file pntd.0011292.s007.pdf]

Universidade Federal da Bahia
Instituto de Saúde Coletiva
Rua Basílio da Gama S/N, Canela - 40.110-040 Salvador – Bahia – Brasil

E-mail: fcosta2001@gmail.com
federico.costa@ufba.br

Salvador, April 17, 2024.

Joseph Vinetz
Editor, PLOS Neglected Tropical Diseases

Claudia Munoz-Zanzi
Guest Editor, PLOS Neglected Tropical Diseases

In re: PNTD-D-23-00442R2

Dear

We thank the reviewers for their thoughtful and constructive comments and suggestions. We are submitting our responses along with a revised manuscript, "Factors associated with differential seropositivity to *Leptospira interrogans* and *Leptospira kirschneri* in a high transmission urban setting for leptospirosis in Brazil", for consideration as a research article to be published in PLOS Neglected Tropical Diseases.

Again, we appreciate your consideration in reviewing our manuscript. Please contact me if there are any questions on the preparation of the revision.

Sincerely,

Federico Costa

Corresponding Author

Instituto de Saúde Coletiva, Salvador, Brazil

Manuscript reference number: PNTD-D-23-00442R2

Date: April 17, 2024.

Response to Reviewer –

Factors associated with differential seropositivity to *Leptospira interrogans* and *Leptospira kirschneri* in a high transmission urban setting for leptospirosis in Brazil

****

It has been increasingly recognized that MAT has limited value for classification. It is widely used and plays an important role in sero-epidemiological studies and serologic surveillance; however, as stated in previous reviews caution is needed when making claims about strain identification and causality. I think the current language in this revised version still emphasizes causality without giving proper context. The local disease ecology may be suitable, better than in other locations, due to may be few circulating strains, but this is not immediately represented in the text.

I believe that if the definition used and assumptions made are properly described and justified in the Methods section, the rest of the analysis is appropriate. Then, in the discussion section, this limitation can be reiterated and the current statement expanded to discuss the impact on the results.

Specific changes:

Line 111: The microscopic agglutination test (MAT) is the reference serological assay for the diagnosis of leptospirosis. This enables identification of the strain involved in the infection. It can also help to indicate the serogroups circulating in a specific area or region, thereby supporting spatiotemporal epidemiological studies and reservoir identification [11].

This sentence explicitly states that MAT is used for strain identification, which is not the case. Regarding serogroup identification, "identification of presumptive serogroup" has been used more recently as a way to represent the uncertainty in such classification.

Response: We appreciate the comment and insert the suggestion in lines (112-115). The changes are highlighted in yellow.

Line 183: Samples were tested against a panel of seven antigens, including five reference strains (WHO Collaborative Laboratory for Leptospirosis, Royal Tropical Institute, Holland): L. kirschneri serovars Cynopteri strain 3522C and Grippothyphosa strain Duyster; L. interrogans serovars Canicola strain H. Utrecht IV and Autumnalis strain Akiyami A; and L. borUTHgpetersenii serovar Ballum strain MUS 127.

State also the serogroups for those strains since this is what MAT resulting are indicating/approximating.

Response: Thank you for this comment and suggestion. Following the editor's suggestion, we added the respective serogroups to the paragraph (lines 185-191).

Line 196: The outcome of seropositive for leptospirosis was defined as seropositivity against serogroups Cynopteri and/or Icterohaemorrhagiae. Line 200: Seropositivity for a specific serogroup was defined as the one with the highest titer.

Explain here the limitation of using MAT for serogroup classification, that it may only give an idea of the presumptive serogroup, and how MAT results were interpreted to produce the outcome for analysis. For example: highest titer, at least 2 titers higher than the titer for any other serogroup, how it was classified if the highest titer was to more than one serogroup, describe how “negative” (the comparison group) was defined, etc.

Response: Thank you for this comment and suggestion. Following the editor’s suggestion, we re-formulated the sentence (lines 197-201 and 204-210).

Line 315: In this paper, we found that L. interrogans serogroup Icterohaemorrhagiae was the main serogroup responsible for leptospirosis cases in Salvador.

This statement needs to be toned down regarding finding that serogroup Icterohaemorrhagiae was the main infecting serogroup. Statement needs to be consistent with the limitation of MAT to identify serogroups. If in the local context of expected few circulating serogroups and the interpretation of results (for example: few individuals had titers to other serogroups, there was little evidence of cross-reactions,]etc. – these results are not shown), then authors can elaborate a bit more about the relative importance of the investigated serogroups.

Response: We appreciate your comment and suggestion. In response to the editor's feedback, we have revised the sentence (lines 326-340 and 284-286) as suggested. Additionally, we have included two supplementary tables (tables S1 and S2) presenting the seropositivity results by serogroup.
