# Supplementary material for: Factors associated with differential seropositivity to Leptospira interrogans and Leptospira kirschneri in a high transmission urban setting for leptospirosis in Brazil
Source: PLoS Negl Trop Dis. 2024 May 17;18(5):e0011292. doi: 10.1371/journal.pntd.0011292 (PMC11139309; doi:10.1371/journal.pntd.0011292)
Supplement: Attachment — Submitted filename: Response to Reviewer_DSO.docx [file pntd.0011292.s005.docx]

Universidade Federal da Bahia

Instituto de Saúde Coletiva

Rua Basílio da Gama S/N, Canela - 40.110-040 Salvador – Bahia – Brasil

E-mail: [fcosta2001@gmail.com](mailto:fcosta2001@gmail.com) [federico.costa@ufba.br](mailto:federico.costa@ufba.br)

Salvador, November 10th, 2023.

Joseph Vinetz

Claudia Munoz-Zanzi

Editors, PLOS Neglected Tropical Diseases

In re: PNTD-D-23-00442

Dear

We thank the reviewers for their thoughtful and constructive comments and suggestions. We are submitting our responses along with a revised manuscript, "Factors associated with differential seropositivity to *Leptospira interrogans* and *Leptospira kirschneri* in a high transmission urban setting for leptospirosis in Brazil", for consideration as a research article to be published in PLOS Neglected Tropical Diseases.

Again, we appreciate your consideration in reviewing our manuscript and please contact me if there are any questions on the preparation of the revision.

Sincerely,

Federico Costa

Corresponding Author

Instituto de Saúde Coletiva, Salvador, Brazil

Manuscript reference number: PNTD-D-23-00442

Date: November 10th, 2023.

Response to Reviewer –

**Factors associated with differential seropositivity to *Leptospira interrogans* and *Leptospira kirschneri* in a high transmission urban setting for leptospirosis in Brazil**

***********************

In addition to addressing the reviewers' questions, in particular regarding methodological and results details in study design and analysis, please address the following:

MAT is the reference serological test but not a gold standard (and not “golden”) which implies perfect sensitivity and specificity. Please change to reference test.

Response: Thank you for this comment and suggestion. We accepted the editor’s suggestion (line 112).

Regarding the use of MAT as the primary method for the study. In Line 90, authors indicate that MAT can indicate presumptive infective serogroup. We generally use that statement as a word of “caution”, to convey that it is a far we should go in terms of attempting to interpret MAT results since MAT cannot identify serogroups and certainly not serovars. The short sentence in the introduction seems to imply that MAT is a tool validated for identifying serogroups and I believe this needs to be justified further in the context of this specific study and application. Presumptively means that there is a lot of room for misclassification which will affect results.

The use of MAT in the specific study in Salvador is fairly unique because of the long-term research and understanding of the local ecology indicating very few circulating strains and serogroups which would facilitate interpretation of MAT results. This would not be applicable to other locations with many circulating strains. Authors do explain the study is examining serologic profiles which is a valuable tool but I recommend expanding that introduction to clearly explain that MAT does not identify serogroups, that presumptive serogroups can be reported under certain assumptions, but it can be useful in some contexts such as population level examination of serologic profiles, in particular to detect temporal or spatial shifts in the profiles. Methods need to clearly specify the assumptions for interpretation of MAT and the discussion should include limitations due to potential MAT misclassifications and missing detection of other strains.

Response: Thank you for this comment and suggestion. Following the editor’s suggestion we have expanded the introduction and discussion to explain the limitations and assumptions for interpretation of strains included in the MAT performed in our study (lines 111-115).

Reviewer's Responses to Questions

**Key Review Criteria Required for Acceptance?**


As you describe the new analyses required for acceptance, please consider the following:

**Methods**
-Are the objectives of the study clearly articulated with a clear testable hypothesis stated?

-Is the study design appropriate to address the stated objectives?

-Is the population clearly described and appropriate for the hypothesis being tested?

-Is the sample size sufficient to ensure adequate power to address the hypothesis being tested?

-Were correct statistical analysis used to support conclusions?

-Are there concerns about ethical or regulatory requirements being met?


Reviewer #1: Overall, the study demonstrates a clear articulation of objectives, employs an appropriate study design, and describes a suitable population. The study recruited a large sample size and employed robust statistical analyses, which adequately support the claims.

Reviewer #2: Why was Pau da Lima surveyed so much later than the other neighborhoods? I do have some concerns about this given that it also makes up roughly half of the total sample size. Table 1 mostly alleviates these concerns, but this should be discussed especially given what occurred between 2018 and 2020, namely COVID-19. Are we sure that these are comparable populations.

Response: We appreciate the reviewers' questions and suggestions.

We appreciate the feedback and concern. First, it is important to point out that all areas studied were selected due to comparable environmental and social characteristics, which make these populations equally susceptible to infectious diseases, such as leptospirosis. The specific variables used to characterize and choose the areas in order to be comparable to Pau da Lima (our primary study site) were: Population density was 12,651 inhabitants residing in an area of 0.46 km2; Elevation; Proportion of head of the household income <1 minimum wage; Proportion of head of the household completed 1° school; incidence of hospitalized leptospirosis.

The initial study did not included Pau da Lima and we identified a notable association between the seropositivity of individuals by the Cynopteri serogroup and the presence of cats in households. However, because of the low prevalence of Cynopteri, the model did not allow us to include significant covariates. Based on this significant correlation, we decided to include in the questionnaire the cohort from the Pau da Lima neighborhood, which we have followed for over 20 years. We hope that these additional clarifications contribute to a better understanding of the reasons behind the methodological decisions adopted and how we seek to address the pertinent concerns raised.

Line 149: How was eligibility determined?

Response: Thank you for this comment. The eligibility of participants was determined based on specific criteria, namely:

Sleep at least 3 nights a week in the house where the study took place;

Be 5 years old or older;

Provide written informed consent, demonstrating willingness to participate in the study after information and clarifications about the research;

In the case of individuals under the age of 18, obtain the written consent of their legal guardians, guaranteeing authorization for their participation.

After verifying that potential participants met these criteria, all eligible and willing individuals were recruited to take part in the research. This approach helps us to ensure that the participants comply with the ethical and legal procedures involved in the research

We have added some of this information to the manuscript to help clarify this issue (lines 173-176).

Line 149: What is meant by “free consent”? Is this informed consent? Also, were eligible individuals offered any incentive to participate?

Response: Thank you for this comment. Yes, it is an informed consent form, that is, it assumes that the participant and/or their legal guardian were free to decide to participate voluntarily or withdraw from the study at any time, without suffering any harm. We changed the term "free consent" to "informed consent" (line 176).

Participants received only the results from some laboratory tests provided by the project as an incentive, with no other form of reward or inducement for participating in the study.

Line 149: How were eligible individuals identified and approached, e.g. door-to-door visits?

Response: Thank you for this comment. In fact, the project field team approached the participants through door-to-door home visits. This approach enabled direct contact with potential participants, ensuring the opportunity to explain in detail the objectives of the study, provide relevant information and clarify any doubts that might arise about the participation process. This entire procedure was carried out in a respectful and ethical manner, safeguarding the individuals' right to privacy and free and informed consent.

Line 151: Please include a sample questionnaire as supplemental information or a citation if it was used previously.

Response: We used this previous manuscript: Khalil H, Santana R, de Oliveira D, Palma F, Lustosa R, Eyre MT, et al. Poverty, sanitation, and leptospira transmission pathways in residents from four Brazilian slums. PLoS Negl Trop Dis. 2021;15: 1–15. doi:10.1371/journal.pntd.0009256.

Please include both the precise starting statistical model and the model after variable selection for both analyses. It is unclear to me which variables were initially included and which were excluded by the stepwise variable selection.

Response: Thank you for this comment and suggestion. The variables initially included are shown in the bivariate s1 table, then those that presented p<0.15 were included in the multivariate model. It is important to clarify that the selection of variables included in the multivariate analysis was based on statistical and researcher criteria (biological plausibility) (lines 247-250).

I have concerns about the initial screen of variables using bivariate analysis and a p<0.15 threshold. Variables that are not statistically significant in bivariate analysis can be in multivariate analysis. It would be better to include all biologically relevant variables and allow the stepwise variable selection to choose, assuming the model will converge with all variables.

Response: Thank you for this comment and suggestion. We chose a conservative p-value threshold of <0.15 for our initial bivariate analysis to be more inclusive of variables that could potentially be important in the multivariate model. We use this approach since we are dealing with a complex system with a wide range of variables across different dimensions (demographic, environmental, socioeconomic) [1]. We are aware that variables that may not be significant (at p < 0.05) in bivariate analysis may become so in a multivariate context. However, our choice of a higher p-value in the initial screening was a trade-off to reduce the number of variables and ensure model convergence, given the large number of variables we started with.

Our approach aligns with methods commonly used in epidemiological studies, where a liberal p-value is often used in bivariate analyses to preselect variables for more complex multivariate models [e.g. 1, 2 ].

1- Victora CG, Huttly SR, Fuchs SC, Olinto MT. The role of conceptual frameworks in epidemiological analysis: a hierarchical approach. Int J Epidemiol. 1997 Feb;26(1):224-7. doi: 10.1093/ije/26.1.224. PMID: 9126524.

2-Felzemburgh RDM, Ribeiro GS, Costa F, Reis RB, Hagan JE, et al. (2014) Prospective Study of Leptospirosis Transmission in an Urban Slum Community: Role of Poor Environment in Repeated Exposures to the *Leptospira* Agent. PLOS Neglected Tropical Diseases 8(5): e2927

The authors mention using a mixed-effect model, but don’t mention a random effect.
Response: Thank you for this comment. The reviewer is correct here, we do not use random effects, as we included community as a fixed effect in the model. We have now corrected that in the text (line 245).

Please include the standard diagnostic plots for all statistical models as supplemental information so that the appropriateness of the models can be determined.
Response: Thank you for this comment and suggestion. In the context of multinomial logistic regression models, traditional diagnostic plots used for linear regression are not directly applicable (such as Q-Q or Cook’s distance plots). However, we conducted other diagnostic checks to assess the appropriateness of our models, including Pearson Chi-Square Goodness-of-Fit tests.

--------------------

**Results**


-Does the analysis presented match the analysis plan?

-Are the results clearly and completely presented?

-Are the figures (Tables, Images) of sufficient quality for clarity?


Reviewer #1: The analysis presented in the manuscript matches the analysis plan described in the methods section. The results are clearly and completely presented in the manuscript.

In Table 1, the study presents three outcomes for *Leptospira* seropositivity: "Icterohaemorrhagiae," "Cynopteri," and "No infection." However, the term "No infection" may not be the most appropriate label in this context. It implies the absence of infection by any serogroups, but the study only used two strains in the MAT to determine seropositivity. Therefore, there is a possibility that the samples could have been infected with serogroups not included in the MAT. It would be helpful if the author could clarify this point or acknowledge it as a potential limitation in the study.

Response: We appreciate the comment and have included this point in the limitation discussion (lines 379-390). We also changed the nomenclature from “No infection” to “Without evidence of seropositivity”. Initially, we used a selection of bacteria representing 8 serogroups of *Leptospira*, chosen based on a hospital surveillance study carried out in a reference hospital for infectious diseases, which identified the most reactive serogroups in the region. However, for association analysis purposes, we focused our efforts on the most prevalent serogroups, namely Copenhageni strain Fiocruz L1-130 and Cynopteri strain 3522C.

It is important to highlight that, although we previously tested a larger battery, 8 serogroups, we recognize that there may be other *Leptospira* serogroups that were not included in the study's TAM assay. However, it is important to point out that our main objective was not focused on the comprehensive evaluation of all serogroups in each area, but on identifying the factors that could be associated with the seropositivity of the main *Leptospira* serogroups that are prevalent in the studied populations.

Reviewer #2: In Table 1: Would “Wall material?” be better described as “Wall Plastered?”

Response: Yes, we agree. Thanks for the suggestion, we corrected the term in the table.

Figure 1: This figure or the data used to create needs a citation.

Response: Thank you for this comment and suggestion. We have inserted the citation in the text (lines 234-240).

Line 237: Where any individuals seropositive for both?

Response: Thank you for this comment. Yes, we identified an overall seropositivity of 9% for both serogroups in all studied areas. We have included this data in the results (line 273).

Line 243: Did any of the neighborhoods have significantly different seropositivity rates from the others before adjusting for other covariates?

Response: Thank you for this comment. The significance rates were added to the table S1.

Line 245: You state that seroprevalence of Icterohaemorrhagiae increased with age group, what precisely do you mean here? And what is the p-value associated with the associated test of significance?

Response: Thank you for this comment. We made some changes to the text to better explain the information (lines 283 –287).

Figure 2:
The axes need labels as do panel A and B.
Are these seropositivity rates as a percentage of total population?
The upper bound of the male 45+ group on the left plot was cut off
Please add labels showing which groups are significantly different from others
Please change gender to sex

Response: We appreciate all the suggestions made by the reviewer. We indicate that they were added throughout the text and figure.

Line 266: What model was used for each analysis? Did the analysis of age and sex shown previously include only those variables or other covariates? What variables were included in this analysis?

Response: Thank you for this comment. All the variables presented in table S1 and from there all the variables that presented p<0.15 were taken to the multivariate model, as described in lines 283-287.

Line 272: What age group was the OR given for and what was the comparator group?

Response: Thank you for this comment. To conduct the univariate and multivariate analyses, we incorporated the age variable as a continuous measure. In order to represent the relative distribution between age and gender, we structured the age variable into age group categories, as shown in Figure 2.

Table 2: What do the dashes denote? Are they variables that were not included in the final model?

Response: Thank you for this comment. The dashes mean that these variables are not part of this model.

--------------------

**Conclusions**


-Are the conclusions supported by the data presented?

-Are the limitations of analysis clearly described?

-Do the authors discuss how these data can be helpful to advance our understanding of the topic under study?

-Is public health relevance addressed?

Reviewer #1: Overall, the authors have presented a comprehensive analysis and discussion of the data, ensuring the conclusions are well-supported. They provide a clear understanding of how these findings can serve as a foundation for future research, emphasizing the significant public health implications of the study.

It would be helpful to expand and elaborate further on the limitation section in the discussion. This could involve addressing any potential biases or confounding factors that may have influenced the results. Additionally, limitations related to the study design, data collection methods, and generalizability of the findings should be acknowledged.

Response: Thanks for the comment. We understand that we need to delve deeper into the limitations of the study, so we address this point further in the lines 379-390.

Reviewer #2: Line 305: If feel like the statement “suggesting that behavioral differences between men and women do not affect exposure” is too strong of a statement given that behavioral differences in men and women were not explored in the analysis. If there are specific covariates that are expected to vary between men and women, these should be discussed and evaluated.

Response: Thank you for this comment. We understand that we need to further elaborate in the issue to support this claim, although no specific analysis on the topic was performed in this study. Previous studies indicate that men and women present differences in seroprevalence that are associated to the activities typically associated to each gender in the study area. In our study areas, the female population is more often responsible for indoor activities such as housekeeping, being caretakers of children and/or other relatives, or economic activities developed from the home. On the other hand, men are more involved with activities that increase their exposure to potential contamination. Such activities include traversing the unpaved and prone-to-water-accumulation pathways in the communities (both more frequently and for longer distances), cleaning and unclogging sewer canals, active management of outdoor environments (such as cleaning or disposing garbage piles, construction work, landscaping), and even recreative activities such as playing sports in open unpaved terrain (lines 330-335).

Line 360: The authors only explored the spatial niches of seroprevalence, not of the reservoirs.

Response: Thank you for this comment. We have updated the sentence (lines 396-398). Our data show different exposure risks and territorial spatial niches for different serogroups, which can potentially indicate different epidemiology or transmission pathways.

--------------------

**Editorial and Data Presentation Modifications?**


Use this section for editorial suggestions as well as relatively minor modifications of existing data that would enhance clarity. If the only modifications needed are minor and/or editorial, you may wish to recommend “Minor Revision” or “Accept”.

Reviewer #1: None.

Reviewer #2: Line 102: I’m not sure what you mean by an “informal accelerated expansion”.

Response: Thank you for this comment. In the sentence presented, "informal accelerated expansion" refers to urban growth or development that occurs in an unplanned manner, without following urban regulations and without the proper formalization of buildings and infrastructure. In other words, it is the disorderly growth of urban areas, often characterized by the construction of housing and infrastructure in an improvised way and without following the norms and standards established by the competent authorities. We changed the phrase to "rapid improvised expansion" (line 126).

Line 190: possible typo – should this read “as complementary analyses to evaluate”.
Line 191: I’m not sure what the last part of the sentence after “with” is referring to.

Response: Thank you for this comment. In observation of the reviewer's considerations for lines 190 and 191, we suggest changing the text, the updated phrase is on the lines 221-224.

Line 319: possible typo – should “maybe because have a” be “maybe due to a”?

Response: We appreciate the reviewer's comments and performed a review. We updated the sentence according to the suggestion made by the reviewer (line 348).

Line 354: suggested rewording – “which might not be possible to understand the true” might be better written as “might make it difficult to understand the true”

Response: We thank the reviewer for the comment and formulated the sentence according to the suggestion made (line 390-391).

--------------------

**Summary and General Comments**


Use this section to provide overall comments, discuss strengths/weaknesses of the study, novelty, significance, general execution and scholarship. You may also include additional comments for the author, including concerns about dual publication, research ethics, or publication ethics. If requesting major revision, please articulate the new experiments that are needed.

Reviewer #1: The main claims of the paper are to identify the factors associated with seropositivity to Leptospira serogroup Icterohaemorrhagiae and serogroup Cynopteri in a high transmission urban setting for leptospirosis in city of Salvador, Brazil, and to analyze the spatial distribution patterns of these serogroups within the study area. These claims are novel and significant as they contribute to understanding the epidemiology of leptospirosis, specifically the risk factors and spatial distribution of different pathogenic serogroups in urban environments. While previous studies have investigated leptospirosis in similar contexts, this study's specific focus on these two serogroups and their associations with demographic, socioeconomic, and environmental factors adds novelty to the field.

Reviewer #2: I thank the author’s for their submission “Factors associated with differential seropositivity to Leptospira interrogans and Leptospira kirschneri in a high transmission urban setting for leptospirosis in Brazil”. Overall, I found the study well designed, the analysis appropriate, and the paper well written. I do believe that the paper could benefit from several revisions. Most importantly, I believe the methods would benefit from significantly more details about the models used as it not currently possible to replicate their results.

--------------------

PLOS authors have the option to publish the peer review history of their article ([what does this mean?](https://journals.plos.org/plosntds/s/editorial-and-peer-review-process#loc-peer-review-history)). If published, this will include your full peer review and any attached files.


If you choose “no”, your identity will remain anonymous but your review may still be made public.


**Do you want your identity to be public for this peer review?** For information about this choice, including consent withdrawal, please see our [Privacy Policy](https://www.plos.org/privacy-policy).

Reviewer #1: No

Reviewer #2: No


Figure Files:
While revising your submission, please upload your figure files to the Preflight Analysis and Conversion Engine (PACE) digital diagnostic tool, https://pacev2.apexcovantage.com. PACE helps ensure that figures meet PLOS requirements. To use PACE, you must first register as a user. Then, login and navigate to the UPLOAD tab, where you will find detailed instructions on how to use the tool. If you encounter any issues or have any questions when using PACE, please email us at figures@plos.org.
 
Data Requirements:
Please note that, as a condition of publication, PLOS' data policy requires that you make available all data used to draw the conclusions outlined in your manuscript. Data must be deposited in an appropriate repository, included within the body of the manuscript, or uploaded as supporting information. This includes all numerical values that were used to generate graphs, histograms etc.. For an example see here: http://www.plosbiology.org/article/info%3Adoi%2F10.1371%2Fjournal.pbio.1001908#s5.
 
Reproducibility:
To enhance the reproducibility of your results, we recommend that you deposit your laboratory protocols in protocols.io, where a protocol can be assigned its own identifier (DOI) such that it can be cited independently in the future. Additionally, PLOS ONE offers an option to publish peer-reviewed clinical study protocols. Read more information on sharing protocols at https://plos.org/protocols?utm_medium=editorial-email&utm_source=authorletters&utm_campaign=protocols

__________________________________________________
In compliance with data protection regulations, you may request that we remove your personal registration details at any time. (Use the following URL: https://www.editorialmanager.com/pntd/login.asp?a=r). Please contact the publication office if you have any questions.
